# Ureteral Obstruction Secondary to an Appendiceal Mucocele: A Case Report and Literature Review

**DOI:** 10.1089/cren.2018.0022

**Published:** 2018-05-01

**Authors:** Melody Djuimo, Liane S. Feldman, Sero Andonian

**Affiliations:** ^1^ Department of Urology, McGill University Health Centre, Montreal, Canada.; ^2^ Department of Surgery, McGill University Health Centre, Montreal, Canada.

**Keywords:** mucocele, appendix, ureteral obstruction, ureteral calculi, management

## Abstract

***Background:*** Ureteral obstruction is rarely caused by extrinsic compression from the appendix. In addition, mucinous neoplasms of the appendix are rare, found incidentally in 0.2%–0.7% of appendectomy specimens.

***Case Presentation:*** We present an unusual case of ureteral obstruction caused by a large appendiceal mucocele. An asymptomatic 53-year-old caucasian male patient, known for recurrent nephrolithiasis, was referred for management of bilateral nephrolithiasis. A noncontrast CT scan found an atrophic kidney with an obstructive 1.8 cm right midureteral stone above a 9 × 4.3 cm appendiceal mucocele compressing the right midureter. Although the impacted ureteral stone was extracted with retrograde ureteroscopy and holmium laser lithotripsy, the appendiceal mucocele was resected by laparoscopic appendectomy. Final pathology analysis revealed an unperforated low-grade appendiceal mucinous neoplasm of 13 cm without lymphovascular invasion. Since the whole mucocele was completely excised, it did not require any further follow-up. Postoperative CT scan demonstrated stone-free status on the right side with residual mild right hydroureteronephrosis. A diuretic renal scintigraphy showed a nonobstructed right kidney with a chronically dilated pelvicaliceal system and a 34% differential function in the right kidney. Follow-up for up to 2 years postoperatively demonstrated that his diuretic renal scan did not show deterioration of the differential renal function, indicating that there was no significant obstruction.

***Conclusion:*** This is the fourth reported case of appendiceal mucocele causing extrinsic ureteral obstruction, and the secondary urinary stasis contributed to formation of a large midureteral stone and cortical renal atrophy. This case demonstrates the importance of the work-up and management of obstructive ureteral stones before definitive management of appendiceal mucoceles.

## Introduction

Ureteral obstruction causes can be classified into two categories: intraluminal and extraluminal. Although ureteral stones, tumors, or clots are the intraluminal causes, benign or malignant lesions arising from adjacent organs represent the extraluminal entities responsible for ureteral obstruction. Among the adjacent organs involved, the appendix is rarely the culprit.^1–3^ Primary tumors of the appendix are uncommon and are often diagnosed after pathologic examination of the appendix.^4^ The most common neoplasm of the appendix is mucinous neoplasm of the appendix, found in 0.2%–0.7% of appendectomy specimens.^1^ We present an unusual case of ureteral obstruction caused by a large appendiceal mucocele. The resulting ureteral obstruction and secondary urinary stasis contributed to formation of a large midureteral stone and renal cortical atrophy.

## Case Report

A 53-year-old male with body mass index of 42 kg/m^2^ was referred for management of bilateral asymptomatic nephrolithiasis. His family history was significant for nephrolithiasis in his brother and sister. He had passed five calcium oxalate stones since the age of 30 years. He was a smoker with no other medical history.

**FIG. 1.**
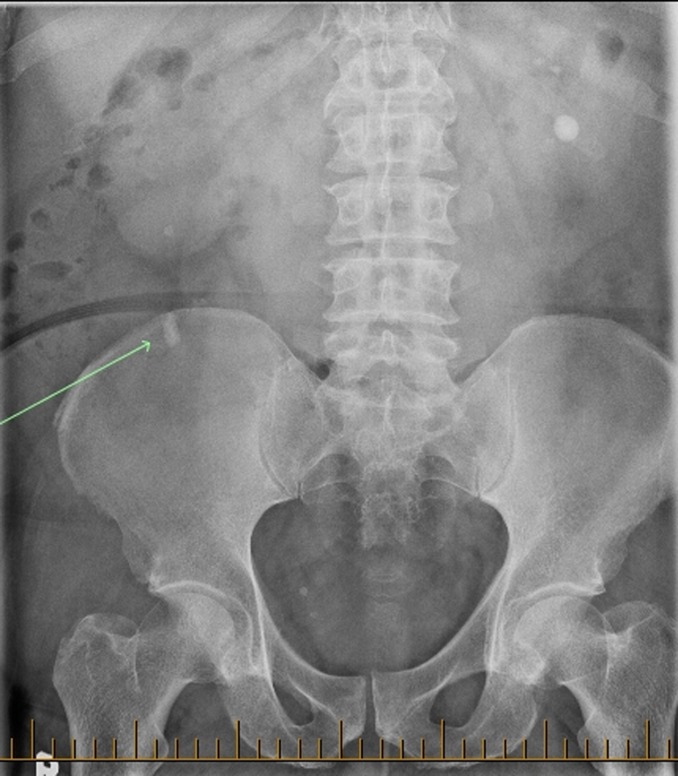
Initial KUB with a right iliac crest calcification (*arrow*) and a left lower pole calcification. KUB = kidney, ureter, and bladder radiograph.

**FIG. 2.**
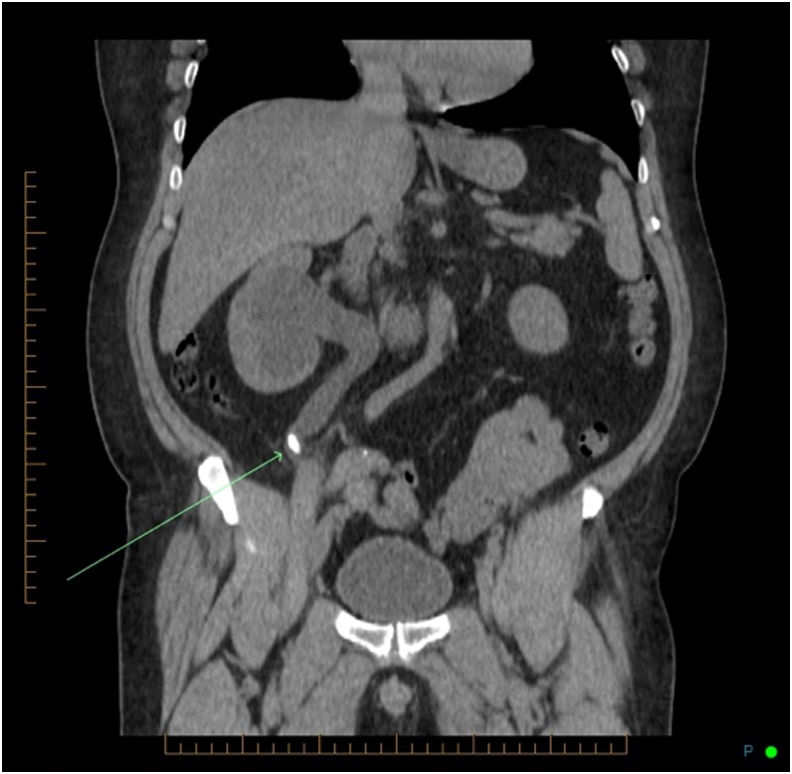
Initial coronal noncontrast CT scan image showing a 1.8 cm midureteral stone, right hydroureteronephrosis proximal to the stone, and thin right renal cortex.

**FIG. 3.**
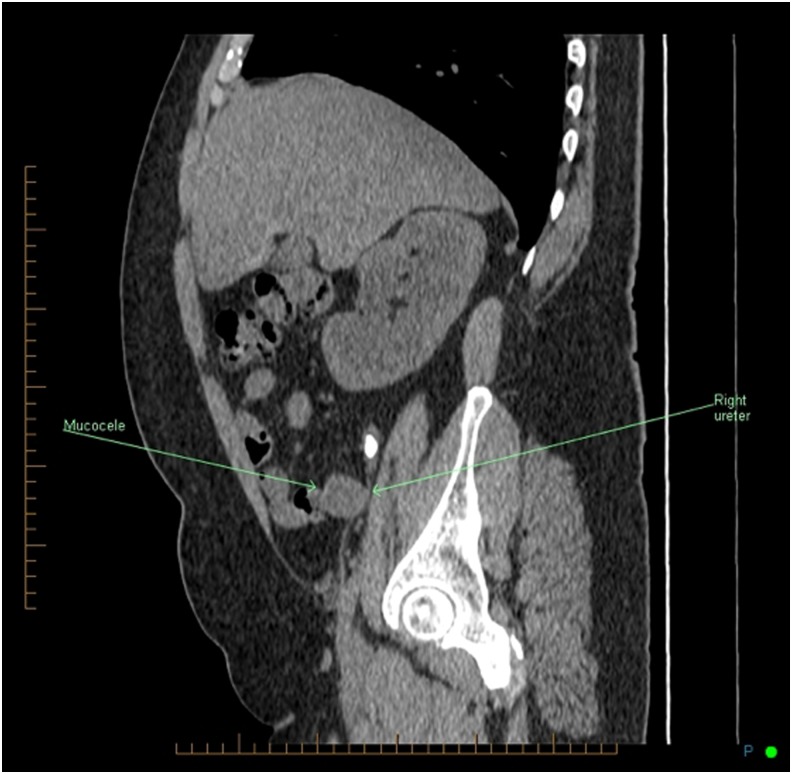
Initial sagittal noncontrast CT scan image showing an appendiceal mucocele compressing the right ureter below the midureteral stone.

**FIG. 4.**
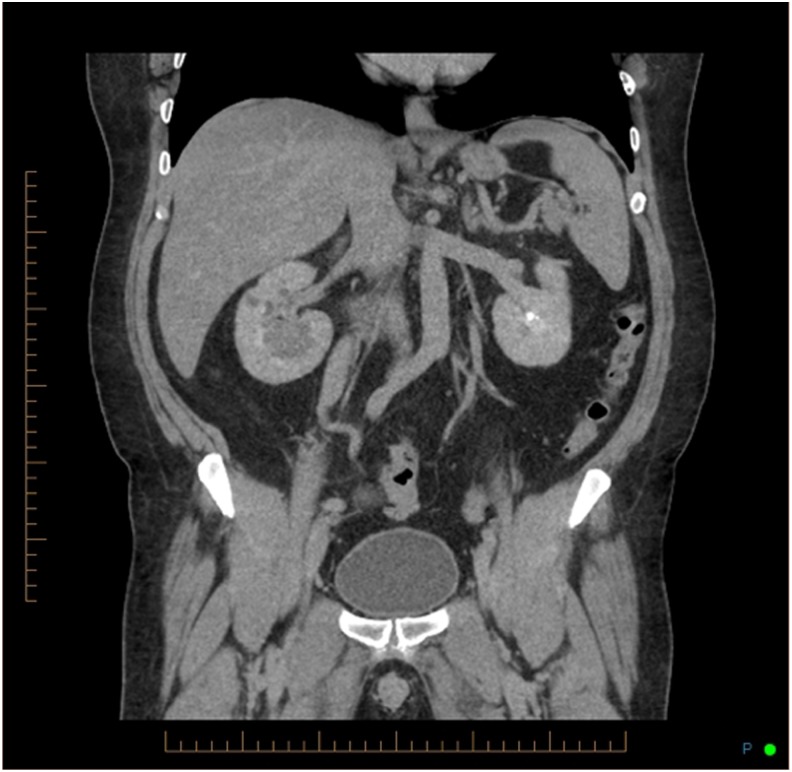
Coronal noncontrast CT scan image postendoscopic removal of the impacted right midureteral stone demonstrating residual chronic right hydroureteronephrosis and a left lower pole caliceal stone.

The initial abdominal plain X-ray (kidney, ureter, and bladder radiograph [KUB]) showed a left lower pole calcification and a 1.5 cm calcification on the iliac crest (Fig. 1). A noncontrast CT of the abdomen and pelvis revealed a 1.8 cm right midureteral stone (1288 HU) with proximal hydroureteronephrosis, a thin right renal cortex, two right lower pole stones 5 mm each (Fig. 2). In addition, a dilated tubular structure measuring 9 × 4.3 cm connected to the cecum and containing a few dependent calcifications was described. This was thought to be an appendiceal mucocele compressing the right mid ureter below the midureteral stone (Fig. 3). On renal scintigraphy, the right kidney was obstructed and contributed to 32% of the global renal function. After a failed attempt of extracorporeal shockwave lithotripsy, right retrograde flexible ureteroscopy (URS) was performed, which revealed a tortuous right distal ureter up to the midureteral stone that was impacted on an inflammatory stricture. In the presence of the stricture and of a tortuous ureter, we thought it was not safe to proceed with laser lithotripsy because of increased risk of ureteral trauma. An indwelling ureteral stent was placed and the patient was brought back for second-look URS and laser lithotripsy. During the second URS 4 weeks later, the impacted midureteral stone was accessed with a semirigid ureteroscope, and the stone was fragmented with Holmium laser energy (1 J, 10 Hz) and the fragments were removed using a zero-tip basket. At this point, a mucous plug was found in the dilated tortuous proximal ureter. A ureteral access sheath was placed, and using a flexible ureteroscope, the lower pole stones were fragmented and basketed out. An indwelling ureteral stent was placed for 1 week. Urine cytology from the ureter showed mucoid material with rare benign urothelial cells. Six weeks later, a triphasic CT scan showed stone-free status on the right side with residual mild right hydroureteronephrosis and a left lower pole caliceal stone (Fig. 4). A diuretic renal scintigraphy showed a nonobstructed right kidney with a chronically dilated pelvicaliceal system and a 34% differential function in the right kidney. Two years later, his diuretic renal scan did not show deterioration of the differential renal function, indicating that there was no significant obstruction. Stone analysis showed 20% calcium oxalate dihydrate, 70% calcium oxalate monohydrate, and 10% carbonate apatite. Metabolic stone evaluation showed adequate urinary volume of 2.1 L, hypercalciuria secondary to hypernatriuria, hyperuricosuria, and hyperoxaluria. After following low salt, low purine, and moderate oxalate diet, his hypercalciuria persisted, he was started on hydrochlorothiazide and amiloride for prophylaxis against recurrence of renal stones.

Simultaneous to the urologic investigations, he was assessed by general surgery for the appendiceal mucocele. Two weeks after the first URS, he underwent colonoscopy, which did not reveal involvement of the cecum; two sessile polyps were found and biopsied with final pathology analysis of tubular adenoma. Two months after his second URS, he underwent laparoscopic appendectomy with stapling at the base of the appendix to include a rim of cecum. Frozen sections indicated negative margins. At the time of laparoscopy, there were no signs of mucinous ascites nor were any signs of peritoneal deposits. The mucocele was not ruptured during manipulation. The final pathology report showed an unperforated low-grade appendiceal mucinous neoplasm of 13 cm without lymphovascular invasion. Since the whole mucocele was completely excised, it did not require any further follow-up.

## Discussion

Appendiceal mucocele is the result of obstruction of the appendiceal orifice with distention of the appendix caused by intraluminal accumulation of mucoid material. The appendix is the most common intra-abdominal location of mucoid collection. It affects more women than men, usually after the age of 50 year. It can be asymptomatic or cause abdominal pain, weight loss, nausea and vomiting, palpable mass, appendicitis, intussusception, ureteral obstruction, and localized rupture or peritoneal spread with acute abdomen. Histologic changes range from benign epithelium to invasive changes of mucinous adenocarcinoma. Treatment is removal of the mucocele with every effort to keep it intact and avoid perforation. This can be accomplished by laparoscopic or open approach. The mesoappendix is removed to obtain lymph node status. A right hemicolectomy is recommended if the base of the appendix is invaded by the tumor or if malignancy is suspected. Here we present a case of a 53-year old gentleman who presented with an asymptomatic appendiceal mucocele causing compression of the right midureter, midureteral impacted 1.8 cm stone above the obstructed ureter, and an atrophic right kidney. After effective extraction of the impacted right midureteral stone with retrograde URS and holmium laser lithotripsy, the appendiceal mucocele was resected by laparoscopic appendectomy.

There are only three reported cases of ureteral obstruction secondary to an appendiceal mucocele. Risher et al. reported the first case of ureteral obstruction by a 6 cm calcified appendiceal mucocele.^1^ Lee reported another case of an ureteral obstruction by a appendiceal mucocele.^2^ Even bilateral ureteral obstruction by mucinous neoplasm of the appendix has been reported.^3^ Urinary stasis resulting from ureteral obstruction caused by the mucocele could lead to urinary tract infection, pyelonephritis, and stone formation. In the present case, the patient did not have any signs of urinary tract infection. Given the fact that there was chronic right hydroureteronephrosis and renal atrophy (Fig. 4), the ureteral obstruction from the appendiceal mucocele and the concomitant midureteral stone are presumed to have been present for several months to years before presentation. In addition, the ureter was tortuous and pushed laterally because of the mucocele, making the interpretation of the plain radiograph KUB calcification on the right iliac crest more difficult. Therefore, in this case, it was important to obtain a noncontrast CT scan, which was used to make the diagnosis of both the obstructing right midureteral stone and the large appendiceal mucocele, causing midureteral extrinsic obstruction (Fig. 3).

## Conclusion

We present a rare case of obstructive uropathy because of an appendiceal mucocele. Although a rare cause of extrinsic ureteral compression, it should be considered in the work-up of ureteral obstruction. A referral to general surgery is recommended for work-up and management of mucinous neoplasms of the appendix once the diagnosis is suggested on axial imaging. This case also demonstrates the importance of managing obstructive ureteral stones before definitive management of appendiceal mucoceles.

## Abbreviations Used

CT: computed tomography
HU: Hounsfield units
KUB: kidney, ureter, and bladder radiograph
URS: ureteroscopy
